# Preschool children's sleep duration and parental depressive symptoms in western China: role of children's mental health and socioeconomic factors

**DOI:** 10.3389/fped.2026.1862244

**Published:** 2026-08-11

**Authors:** Jiali Du, Xi Zhang, Wei He, Mingyue Duan

**Affiliations:** 1 College of Education Science, Yan'an University, Yan’an, Shaanxi, China; 2 Department of Clinical Laboratory, Xi'an Children’s Hospital, Affiliated Children’s Hospital of Xi'an Jiaotong University, National Regional Children’s Medical Center (Northwest), Xi'an, Shaanxi, China

**Keywords:** mental health outcomes, parental depressive symptoms, preschool children, sleep duration, total difficulties, western China

## Abstract

**Background:**

Preschool children's insufficient sleep is a public health concern in underdeveloped western China, yet its association with parental depressive symptoms remains unclear, particularly whether this relationship varies by socioeconomic context. This study aims to examine this association, explore whether child mental health may partly account for it, and identify vulnerable subgroups.

**Methods:**

This cross-sectional study recruited 21,366 parent-child dyads from 189 preschools in western China. Parent-reported children's sleep duration was categorized into three groups: 10–13 h/d (reference), 8–9 h/d, and <8 h/d. Parental depressive symptoms and children's mental health outcomes (total difficulties) were assessed using the Center for Epidemiological Studies Depression Scale (CES-D) and the Strengths and Difficulties Questionnaire (SDQ), respectively. Multivariable logistic regression was used to examine the association between children's sleep duration and parental depressive symptoms, and subgroup analyses were performed to assess effect modification by demographic and socioeconomic factors. Indirect effect analysis was conducted to determine the extent to which children's total difficulties account for this association.

**Results:**

In fully adjusted model, a clear graded relationship was observed that parents of children sleeping 8–9 h/d had 43% higher odds of elevated parental depressive symptoms (OR = 1.43, 95% CI: 1.30–1.56, *P* < 0.001), while parents of children sleeping <8 h/d had 2.6-fold higher odds (OR = 2.61, 95% CI: 2.13–3.19, *P* < 0.001) compared with the 10–13 h/d reference group. Subgroup analyses revealed that the strongest associations were found among mortgaged homeowners (OR = 4.21, 95% CI: 2.52–7.04, *P* < 0.001), urban Hukou registrants (OR = 3.73, 95% CI: 2.62–5.32, *P* < 0.001), and ever-smokers (OR = 4.50, 95% CI: 2.84–7.12, *P* < 0.001) for the <8 h/d group. Indirect effect analyses showed that children's total difficulties accounted for 34.3% (95% CI: 25.1%–47.9%) of the association for children sleeping 8–9 h/d, and 39.4% (95% CI: 30.5%–52.9%) for those sleeping <8 h/d.

**Conclusion:**

Shorter sleep duration in preschool children is associated with an increased risk of parental depressive symptoms, and this association is partially accounted for by children's mental health. Future high-quality cohort studies are needed to explore this topic in greater detail.

## Introduction

Sleep disturbances among children represent a growing global public health issue, with prevalence rates continuing to rise worldwide (1). In China, large-scale epidemiological studies have recently documented a concerning increase in pediatric sleep disturbances, affecting nearly 30%–40% of children (2). Notably, these challenges are particularly pronounced in western China, where a meta-analysis reported the highest prevalence of sleep problems (47.4%) among all regions, compared with 34.0% nationally and 30.4% in southern China (3). Indeed, prior research has linked socioeconomic disadvantage to poorer child sleep outcomes (4), with mechanisms including pre-sleep worries and environmental disruptions (5), and longitudinal evidence further indicates that lower parental socioeconomic status predicts increases in children's sleep problems (6). Despite the breadth of research on qualitative sleep disturbances, studies quantifying sleep duration particularly in socioeconomically vulnerable regions such as western China remain scarce, impeding the development of targeted interventions addressing insufficient sleep.

Adequate sleep is foundational to children's neurocognitive and psychosocial development, supporting learning, memory, executive function, and social competence (7). Nevertheless, insufficient sleep has become increasingly common, yet remains underrecognized, with secular declines in children's sleep duration (8). Current guidelines [e.g., US National Sleep Foundation (NSF) and American Academy of Sleep Medicine (AASM)] consistently recommend 10–13 h of daily sleep for preschoolers, while noting less than 8 h as detrimental (9, 10). Alarmingly, 25%–40% of children experience insufficient sleep before school age (11), elevating risks for mental health problems (12).

Research consistently demonstrates associations between children's sleep disturbances and parental mental health. Cross-sectional evidence shows that frequent night wakings in children are associated with compromised maternal sleep quality, which is linked to increased depressive symptoms (13). Additionally, maternal depressive symptoms are associated with various sleep disturbances in preschoolers (14, 15). Longitudinal evidence further indicates that maternal history of mental disorders predicts increases in children's sleep problems from ages 3 to 6 (6). The relationship extends beyond maternal influence, as paternal depressive symptoms have also been linked to fewer sleep minutes among children (16). These findings point to a complex and dynamic relationship between sleep and mental health within families (17). Given that various sleep disturbances often co-occur, and cumulatively reduce total sleep time (18), the specific role of shorter sleep duration as a potential key factor in family well-being warrants further investigation.

Extensive evidence has established a robust association between shorter sleep duration and mental health problems in young children. A cross-sectional study documents inverse relationships between sleep duration and internalizing symptoms (19), while prospective studies find that insufficient sleep during the preschool period predicts poorer behavioral and social-emotional outcomes at later ages (20, 21). Children's mental health difficulties, in turn, have been linked to parental well-being. Cross-sectional evidence demonstrates associations between children's mental health and parental depressive symptoms (22), including one recent study specifically in preschool-aged children (23). Longitudinal studies have demonstrated that maternal depressive symptoms influence persistent emotional and behavioral difficulties in preschool children (24) and increase the risk of externalizing and internalizing disorders (25). Moreover, bidirectional associations are found between child externalizing problems and maternal depressive symptoms across development (26). Collectively, these findings suggest that child mental health may account for the association between shorter sleep duration and parental depression.

Thus, we hypothesize that children's mental health may play an indirect role in the association between sleep duration and parental depressive symptoms. Notably, socioeconomic factors may not only be associated with children's sleep directly, but also moderate the association between children's sleep and parental mental health. Previous research has shown that the links between children's sleep and family psychosocial functioning are stronger in lower socioeconomic status families (27), and household income has also been found to moderate the relation between maternal depressive symptoms and children's sleep variability (28). These findings suggest that socioeconomic disadvantage may amplify the association between children's sleep and parental depressive symptoms, yet research on such effect modification remains limited. Therefore, this cross-sectional study aims to (1) examine the association between children's sleep duration and parental depressive symptoms, (2) assess whether demographic and socioeconomic factors modify this association, and (3) investigate the proportion of this association accounted for by child mental health.

## Methods

### Study design and participants

This cross-sectional study is a secondary analysis of data from a large-scale epidemiological survey conducted in western China. The original survey employed a stratified cluster random sampling method to recruit preschool children aged 3–6 years from a non-capital city. All 13 administrative districts and counties in this city were included, with 189 public preschools randomly selected proportional to the regional child population density. Data were collected between February 28 and March 5, 2025, through a parent-reported survey that was coordinated by the local Education Bureau and administered via the preschools. Questionnaires were distributed and collected from parents through the preschools, with a 2- to 3-day completion window. The questionnaire was completed by the primary parent (i.e., the parent primarily responsible for the child's daily care), who reported on their own depressive symptoms and health behaviors, as well as their child's sleep, mental health and relevant covariates (Supplementary Table S1).

The original sample comprised 25,017 parent-child dyads; after excluding responses from non-parent caregivers (*n* = 2,408), the remaining 22,609 dyads constituted the eligible sample. Among these, 1,243 (5.50%) had missing or implausible child or parent age data and were excluded from the final analytical sample, resulting in 21,366 dyads for the present study (Supplementary Figure S1).

The study protocol was approved by the Ethics Committee of the Affiliated Children's Hospital of Xi’an Jiaotong University (No. 20250225-21) and conducted in accordance with the Declaration of Helsinki. Written informed consent was obtained from all participating parents or legal guardians.

### Assessment of sleep duration

Parent-reported children's sleep duration was assessed using the question: “On average, how long did your child sleep per day (including daytime and nighttime sleep) during the past month?” Responses were collected in the following categories: <8, 8–9, 10–13, and ≥14 h per day (h/d). Based on established recommendations (9), 10–13 h/d was used as the reference. As no respondents selected ≥14 h/d, analyses focused on the remaining three categories.

### Assessment of parental depressive symptoms

Parental depressive symptoms were assessed using the Center for Epidemiological Studies Depression Scale (CES-D), developed by Radloff in 1977 and widely used to measure depressive symptoms in the general population (29). The full version of the CES-D contains 20 items evaluating the frequency of symptoms experienced during the past week, such as depressed mood, feelings of worthlessness and hopelessness, loss of energy, as well as sleep and appetite disturbances (30). Respondents rate each item on a 4-point Likert scale, and the total scores range from 0 to 60, with higher scores indicating greater depressive symptom severity. A cutoff score of ≥16 was used to identify elevated depressive symptoms, as recommended in previous validation studies (31). The Chinese version of the CES-D has demonstrated good reliability and validity in community-based populations in China, including rural settings (32, 33). In this sample, Cronbach's *α* was 0.85, consistent with previous reports (34).

### Assessment of total difficulties

Children's total difficulties score (TDS) was assessed using the Strengths and Difficulties Questionnaire (SDQ) (35, 36), a widely used and validated screening tool for detecting psychological difficulties in children. This 25-item instrument employs a 3-point scale across five domains: emotional symptoms, conduct problems, hyperactivity/inattention, peer relationship problems, and prosocial behaviors. The TDS is calculated as the sum of the first four subscales, with higher scores denoting greater problems (range 0–40), and a score above cutoff of 14 indicates risk for behavioral and emotional problems (37). The Chinese version of the SDQ has demonstrated acceptable psychometric properties for children aged 3–17 years (37). In this sample, Cronbach's α was 0.70. For descriptive purposes, the TDS was dichotomized using the cutoff, while continuous scores were used in the primary analyses to preserve statistical power.

### Covariate definitions

Demographic and socioeconomic characteristics were collected during initial recruitment, including child and parental age and sex, family structure (single-child status and marital status), socioeconomic status (family annual income, education level, employment status, housing situation and Hukou status), and parental health behaviors (smoking and alcohol use history). Binary variables included single-child status (yes/no), parental employment (employed/unemployed), smoking history (ever-smoker: ex- or current smoker/never-smoker), and alcohol use history (ever-drinker: ex- or current drinker/never-drinker). Family annual income was divided into low-income level (<30,000 CNY), middle-income level (30,000–99,000 CNY), and high-income level (≥100,000 CNY). Parental education level was classified as junior high school or less, high school or junior college, and university degree or above. Housing situation was categorized as owned outright, owned with mortgage, renting, or cohabiting with relatives. Due to limited sample sizes in other categories, marital status was dichotomized into married vs. non-married (cohabitating, divorced, separated, widowed, never-married). Hukou (household registration in China) was categorized as urban or rural. Hukou status was used instead of habitual residence because it captures institutionalized disparities in access to healthcare, social welfare, and public services—factors that are not reflected by current residence alone (38).

### Statistical analysis

Continuous variables are presented as means ± standard deviations, and categorical variables as percentages (n, %). For baseline comparisons, children were stratified by parental depressive symptoms (CES-D ≥16 vs. <16). Between-group differences were assessed using the Mann–Whitney U test for continuous variables and Fisher's exact test for categorical variables.

Multivariable logistic regression was used to analyze the associations between children's sleep duration and parental depressive symptoms, as well as between children's total difficulties and parental depressive symptoms, with results reported as odds ratios (OR) with 95% confidence intervals (95% CI). Multivariable linear regression was performed to examine the association between children's sleep duration and total difficulties, with findings presented as regression coefficients (β) and 95% CI. Three hierarchical models were fitted: Model I (unadjusted), Model II (adjusted for child/parental age and gender), and Model III (fully adjusted for all covariates listed in Covariates section). Effect modification by demographic and socioeconomic factors was evaluated using subgroup and interaction tests. For each stratification variable, a product term was included in the logistic regression model to test for statistical interaction.

We conducted indirect effect analysis to explore whether children's total difficulties account for part of the association between insufficient sleep and parental depressive symptoms. Given the cross-sectional design, this analysis is exploratory and does not imply causality. Using the R mediation package, nonparametric bootstrapping with 1,000 resamples were applied to estimate the indirect effect, direct effect, and total effect (39). Effects are reported as β coefficients with 95% CIs, with statistical significance determined by CIs excluding zero. The proportion of the association accounted for by children's total difficulties was calculated as (indirect effect/total effect) × 100%.

To assess multicollinearity, variance inflation factors (VIF) were calculated for all independent variables in the fully adjusted models (Model III), including sleep duration, total difficulties, and all covariates listed in the Covariates section. All VIF values were below the threshold of 5 (range: 1.0–2.4), indicating no substantial multicollinearity issues (Supplementary Table S2).

The ICC for the primary outcome (CES-D) was estimated using a null model with kindergarten as a random intercept. The ICC was 0.0016, indicating negligible clustering at the kindergarten level (Supplementary Table S3). Using the standard formula for comparing two independent proportions (40) and accounting for the design effect (41), the required sample size was substantially exceeded by our final analytical sample (*N* = 21,366), confirming adequate statistical power.

Missing data were limited to child age (*n* = 1,243, 5.50% of the eligible sample). To handle missing values and assess the robustness of our findings, we performed multiple imputation using chained equations (MICE) with 5 imputed datasets (42). The imputation model included all covariates used in the primary analyses, as well as the outcome (parental depressive symptoms) and key predictor (children's sleep duration). For each imputed dataset, the regression models (main effect and indirect effect) were fitted, and the estimates were combined using Rubin's rules (43). Sensitivity analyses were conducted by comparing the results from the imputed datasets with those from the complete-case analysis.

All analyses used R software 4.2.2 (http://www.R-project.org, R Foundation) and EmpowerStats 3.0 (X&Y Solutions, Inc., Boston, MA; http://www.empowerstats.com), with statistical significance defined as two-tailed *P* < 0.05.

## Results

### Demographic characteristics

This study included 21,366 parent-child dyads, of which 3,372 (15.78%) were identified as at risk for elevated depressive symptoms (CES-D ≥ 16) (Table 1). Statistically significant differences were observed for most baseline characteristics (all *P* < 0.001). Compared with parents in normal CES-D group, those at-risk had lower educational attainment, higher unemployment rates, and were more likely to be unmarried (all *P* < 0.001). Their households more frequently reported low income, rural Hukou, and residential instability. Children of parents at-risk had higher total difficulties scores and were more likely to sleep less than 10 h per day. Detailed comparisons by sleep duration are provided in the (Supplementary Table S4).

**Table 1:** Descriptive statistics of study participants (*N* = 21,366).

Characteristic	Mean ± SD/ *N* (%)			*P-*value
	Total sample (*N* = 21,366)	CES-D normal (*N* = 17,994, 84.22%)	CES-D at-risk (*N* = 3,372, 15.78%)	
Child gender				0.471
Boys	11,062 (51.77%)	9,297 (51.67%)	1,765 (52.34%)	
Girls	10,304 (48.23%)	8,697 (48.33%)	1,607 (47.66%)	
Child age (years)	4.82 ± 0.89	4.80 ± 0.89	4.90 ± 0.87	<0.001
Primary parent gender				0.425
Men	5,108 (23.91%)	4,320 (24.01%)	788 (23.37%)	
Women	16,258 (76.09%)	13,674 (75.99%)	2,584 (76.63%)	
Primary parent age (years)	34.75 ± 4.56	34.71 ± 4.54	34.95 ± 4.70	0.007
Single-child status				<0.001
Yes	6,148 (28.77%)	5,329 (29.62%)	819 (24.29%)	
No	15,218 (71.23%)	12,665 (70.38%)	2,553 (75.71%)	
Marital status				<0.001
Married	20,720 (96.98%)	17,522 (97.38%)	3,198 (94.84%)	
Non-married	646 (3.02%)	472 (2.62%)	174 (5.16%)	
Family annual income, in thousands, CNY				<0.001
<30	8,619 (40.34%)	6,925 (38.49%)	1,694 (50.24%)	
30–99	8,619 (40.34%)	7,349 (40.84%)	1,270 (37.66%)	
≥100	4,128 (19.32%)	3,720 (20.67%)	408 (12.10%)	
Parental education level				<0.001
Junior high school or less	5,741 (26.87%)	4,555 (25.31%)	1,186 (35.17%)	
High school or junior college	9,476 (44.35%)	8,092 (44.97%)	1,384 (41.04%)	
University bachelor or above	6,149 (28.78%)	5,347 (29.72%)	802 (23.78%)	
Parental employment status				<0.001
Employed	15,642 (73.21%)	13,463 (74.82%)	2,179 (64.62%)	
Unemployed	5,724 (26.79%)	4,531 (25.18%)	1,193 (35.38%)	
Housing situation				<0.001
Owned outright	11,577 (54.18%)	10,101 (56.14%)	1,476 (43.77%)	
Owned with mortgage	3,391 (15.87%)	2,802 (15.57%)	589 (17.47%)	
Renting	4,412 (20.65%)	3,510 (19.51%)	902 (26.75%)	
Cohabiting with relatives	1,986 (9.30%)	1,581 (8.79%)	405 (12.01%)	
Hukou status				<0.001
Urban	7,116 (33.31%)	6,132 (34.08%)	984 (29.18%)	
Rural	14,250 (66.69%)	11,862 (65.92%)	2,388 (70.82%)	
Smoking history				0.517
Ever-smoker	3,179 (14.88%)	2,665 (14.81%)	514 (15.24%)	
Never-smoker	18,187 (85.12%)	15,329 (85.19%)	2,858 (84.76%)	
Alcohol use history				<0.001
Ever-drinker	3,508 (16.42%)	2,870 (15.95%)	638 (18.92%)	
Never-drinker	17,858 (83.58%)	15,124 (84.05%)	2,734 (81.08%)	
Sleep duration (h/d)				<0.001
10–13	6,140 (28.74%)	5,420 (30.12%)	720 (21.35%)	
8–9	14,657 (68.60%)	12,175 (67.66%)	2,482 (73.61%)	
<8	569 (2.66%)	399 (2.22%)	170 (5.04%)	
Total difficulties score				<0.001
Continuous	10.13 ± 5.01	9.53 ± 4.78	13.30 ± 5.05	<0.001
TDS ≤14	17,388 (81.38%)	15,360 (85.36%)	2,028 (60.14%)	
TDS >14	3,978 (18.62%)	2,634 (14.64%)	1,344 (39.86%)	

Mea*n* ± SD for continuous variables: the *P* value was calculated by Mann–Whitney U test; (%) for categorical variables: the *P* value was calculated by Fisher's exact tests.

SD, standard deviation; CNY, Chinese Yuan; h/d, hours/day; TDS, total difficulties score.

### Associations between children's sleep duration and parental depressive symptoms

Table 2 presents the associations between children's sleep duration and parental depressive symptoms across three progressive adjustment models. A clear graded relationship was observed across sleep duration categories and this pattern remained evident across all three models. In the unadjusted Model I, parents of children sleeping 8–9 h/d had 53% higher odds of elevated parental depressive symptoms (OR = 1.53, 95% CI: 1.40–1.68, *P* < 0.001), while parents of children sleeping <8 h/d had 221% higher odds (OR = 3.21, 95% CI: 2.64–3.90, *P* < 0.001) compared with the 10–13 h/d reference group. After adjusting for child/parental age and gender in Model II, the associations remained similar (8–9 h/d: OR = 1.51, 95% CI: 1.38–1.65, *P* < 0.001; <8 h/d, OR = 3.12, 95% CI: 2.57–3.80, *P* < 0.001). Further adjustment for all covariates in Model III slightly attenuated the estimates, but the graded pattern persisted (8–9 h/d: OR = 1.43, 95% CI: 1.30–1.56, *P* < 0.001; <8 h/d, OR = 2.61, 95% CI: 2.13–3.19, *P* < 0.001).

**Table 2:** Multivariate logistic regression results for the association between children's sleep duration and parental depressive symptoms.

Sleep duration (h/d)	Model I		Model II		Model III	
	OR (95% CI)	*P*-Value	OR (95% CI)	*P-*Value	OR (95% CI)	*P-*Value
10–13	1.0 [Reference]		1.0 [Reference]		1.0 [Reference]	
8–9	1.53 (1.40, 1.68)	<0.001	1.51 (1.38, 1.65)	<0.001	1.43 (1.30, 1.56)	<0.001
<8	3.21 (2.64, 3.90)	<0.001	3.12 (2.57, 3.80)	<0.001	2.61 (2.13, 3.19)	<0.001

Model I adjusted for none; Model II adjusted for child/parental age and gender; Model III adjusted for child/parental age and gender, single-child status, marital status, family annual income, education level, employment status, housing situation, Hukou status, smoking and alcohol use history.

OR, odds ratio; CI, confidence interval; h/d, hours/day.

### Association between children's sleep duration and total difficulties

Linear regression analysis showed a robust association between children's sleep duration and total difficulties (Table 3). After full adjustment for all covariates, children sleeping 8–9 h/d had a 0.75-point higher total difficulties score (*β* = 0.75, 95% CI: 0.61–0.90, *P* < 0.001), while those sleeping <8 h/d had a 3.12-point higher score (*β* = 3.12, 95% CI: 2.70–3.55, *P* < 0.001), compared with the 10–13 h/d reference group.

**Table 3:** Multivariate linear regression results for the association between children's sleep duration and total difficulties.

Sleep duration (h/d)	Model I		Model II		Model III	
	*β* (95% CI)	*P*-Value	β (95% CI)	*P-*Value	β (95% CI)	*P-*Value
10–13	0 [Reference]		0 [Reference]		0 [Reference]	
8–9	0.91 (0.77, 1.06)	<0.001	0.96 (0.81, 1.11)	<0.001	0.75 (0.61, 0.90)	<0.001
<8	3.69 (3.26, 4.12)	<0.001	3.74 (3.31, 4.16)	<0.001	3.12 (2.70, 3.55)	<0.001

Model I adjusted for none; Model II adjusted for child/parental age and gender; Model III adjusted for child/parental age and gender, single-child status, marital status, family annual income, education level, employment status, housing situation, Hukou status, smoking and alcohol use history.

OR, odds ratio; CI, confidence interval; h/d, hours/day.

### Association between children's total difficulties and parental depressive symptoms

Logistic regression analysis showed a strong association between children's total difficulties and parental depressive symptoms (Table 4). After full adjustment in Model III, each one-unit increase in TDS was associated with a 15% increase in the odds of elevated parental depressive symptoms (OR = 1.15, 95% CI: 1.14–1.16, *P* < 0.001). Using the established cutoff, parents of children at risk (TDS >14) for behavioral and emotional problems had 263% higher odds of elevated depressive symptoms (OR = 3.63, 95% CI: 3.34–3.94, *P* < 0.001) compared with the normal group (TDS ≤14).

**Table 4:** Multivariate logistic regression results for the association between children's total difficulties and parental depressive symptoms.

Total difficulties score	Model I		Model II		Model III	
	OR (95% CI)	*P*-Value	OR (95% CI)	*P-*Value	OR (95% CI)	*P-*Value
Continuous	1.15 (1.15, 1.16)	<0.001	1.16 (1.15, 1.17)	<0.001	1.15 (1.14, 1.16)	<0.001
TDS ≤14	1.0 [Reference]		1.0 [Reference]		1.0 [Reference]	
TDS >14	3.86 (3.57, 4.19)	<0.001	3.95 (3.64, 4.28)	<0.001	3.63 (3.34, 3.94)	<0.001

Model I adjusted for none; Model II adjusted for child/parental age and gender; Model III adjusted for child/parental age and gender, single-child status, marital status, family annual income, education level, employment status, housing situation, Hukou status, smoking and alcohol use history.

OR, odds ratio; CI, confidence interval; h/d, hours/day.

### Subgroup analyses

**Figure 1:**
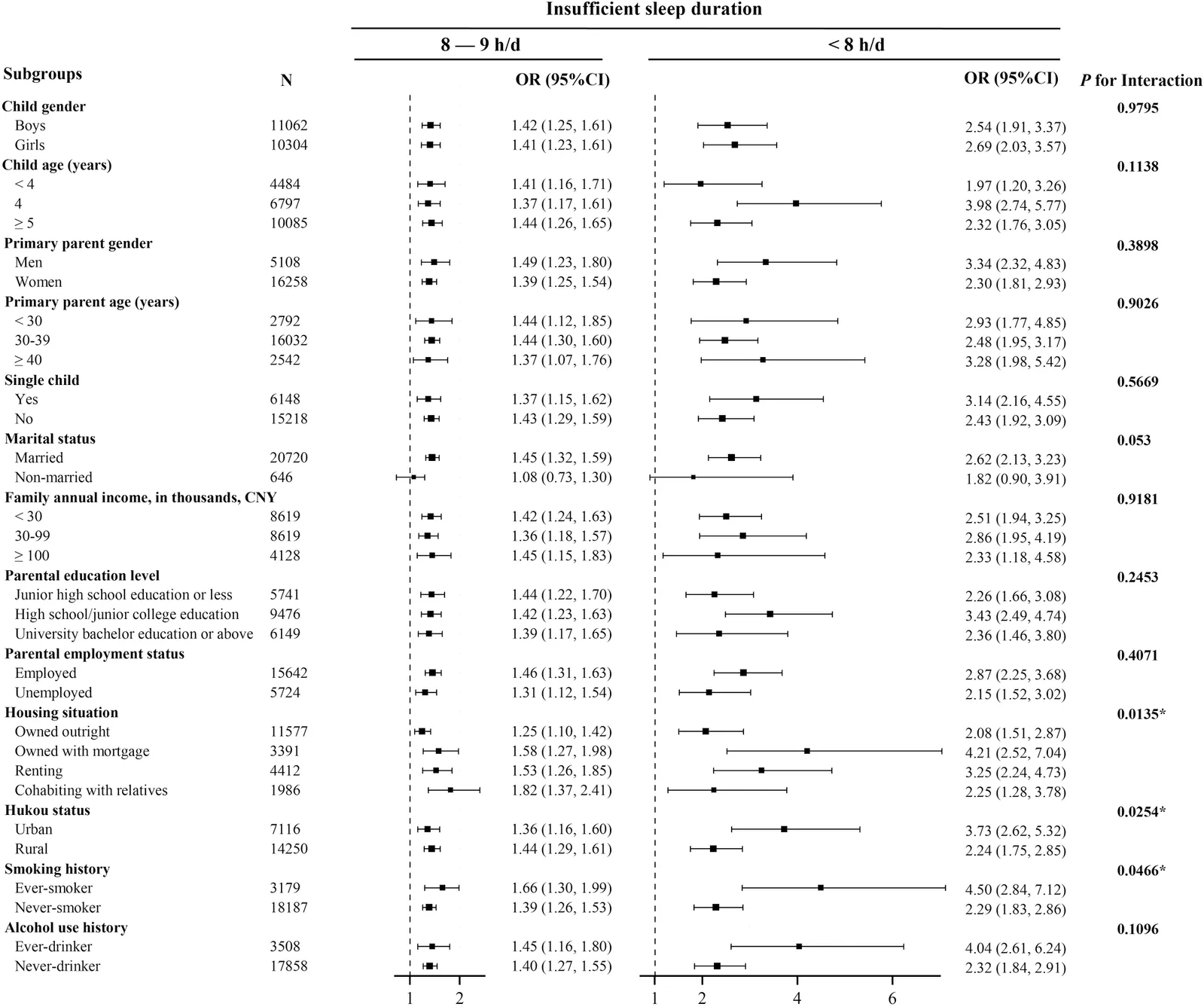
Subgroup analyses of the association between children's sleep duration and parental depressive symptoms. Sleep duration of 10–13 h/d was set as reference. Adjusted for child/parental age and gender, single-child status, marital status, family annual income, education level, employment status, housing situation, Hukou status, smoking and alcohol use history. In each case, the model was not adjusted for the stratification variable itself. OR, odds ratio; CI, confidence interval; h/d, hours/day; CNY, Chinese Yuan.

Subgroup analyses demonstrated consistent associations between children's sleep duration and parental depressive symptoms across most covariates after full adjustment (*P* for interaction >0.05) (Figure 1). Notably, significant effect modification was identified for housing situation (*P* for interactio*n* = 0.0135), Hukou status (*P* for interaction = 0.0254) and parental smoking history (*P* for interaction = 0.0466). For the <8 h/d group, the strength of the association varied progressively across housing situations: the strongest association was found among mortgaged homeowners (OR = 4.21, *P* < 0.001), followed by renters (OR = 3.25, *P* < 0.001), those cohabiting with relatives (OR = 2.25, *P* < 0.001), and outright homeowners (OR = 2.08, *P* < 0.001). Likewise, urban Hukou registrants exhibited stronger associations than rural registrants (OR = 3.73 vs. 2.24, *P* *<* 0.001), and ever-smokers showed substantially elevated risk compared with never-smokers (OR = 4.50 vs. 2.29, *P* < 0.001).

### Indirect effect analysis

**Figure 2:**
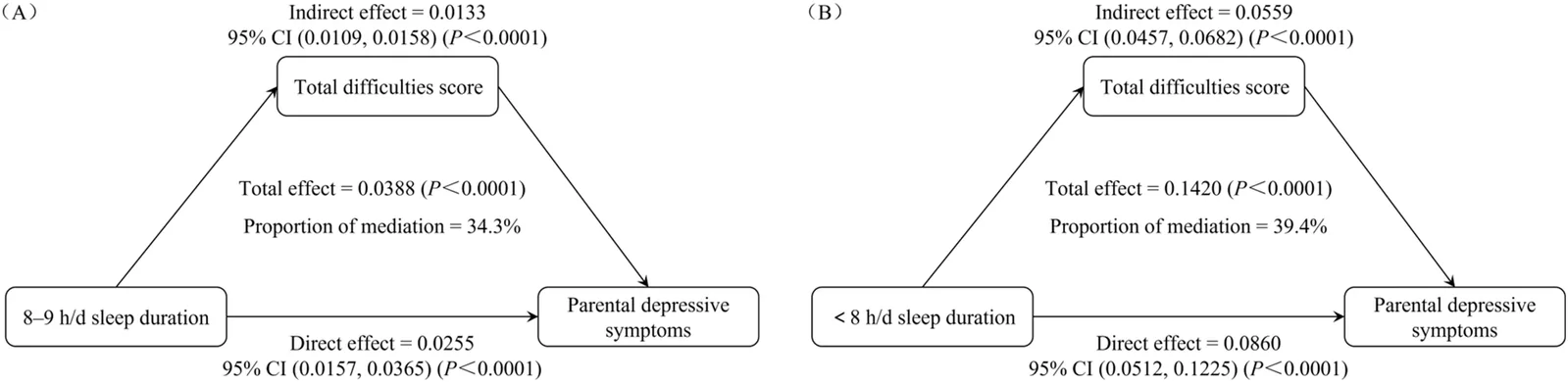
Indirect effect analysis of children's sleep duration on parental depressive symptoms through children's total difficulties. **(A)** 8–9 h/d sleep duration (vs. 10–13 h/d). **(B)** <8 h/d sleep duration (vs. 10–13 h/d). All effects were estimated using nonparametric bootstrap with 1,000 resamples and adjusted for all covariates.

Indirect effect analysis was conducted to examine whether children's total difficulties accounted for part of the association between children's sleep duration and parental depressive symptoms. For the 8–9 h/d group, the average indirect effect was 0.0133 (95% CI = 0.0109–0.0158, *P* < 0.001), accounting for 34.3% (95% CI = 25.1%–47.9%) of the total effect. For the < 8 h/d group, the average indirect effect was 0.0559 (95% CI: 0.0457–0.0682, *P* *<* *0.001*), accounting for 39.4% (95% CI: 30.5%–52.9%) of the total effect (Table 5; Figure 2).

**Table 5:** Indirect effect analysis of children's sleep duration on parental depressive symptoms through children's total difficulties.

Effect	8–9 h/d sleep duration		<8 h/d sleep duration	
	Estimate (95% CI)	*P*-Value	Estimate (95% CI)	*P-*Value
Total effect	0.0388 (0.0290–0.0496)	<0.001	0.1420 (0.1037–0.1793)	<0.001
Indirect effect (average)	0.0133 (0.0109–0.0158)	<0.001	0.0559 (0.0457–0.0682)	<0.001
Direct effect (average)	0.0255 (0.0157–0.0365)	<0.001	0.0860 (0.0512–0.1225)	<0.001
Proportion accounted for (average)	34.3% (25.1%–47.9%)	<0.001	39.4% (30.5%–52.9%)	<0.001

Sleep duration of 10–13 h/d was set as reference. Adjusted for child/parental age and gender, single-child status, marital status, family annual income, education level, employment status, housing situation, Hukou status, smoking and alcohol use history.

CI, confidence interval.

### Sensitivity analyses

To assess the robustness of our findings to missing covariate data, we performed multiple imputation for child and parent age (the variables with missing values). The pooled estimates from the five imputed datasets were consistent with those from the complete-case analysis. For the main regression model, the graded association remained significant (8–9 h/d: pooled OR = 1.41, 95% CI = 1.29–1.54, *P* < 0.001; <8 h/d: pooled OR = 2.56, 95% CI = 2.13–3.07, *P* < 0.001) (Supplementary Table S5). For the indirect effect analysis, the proportion of the association accounted for by children's total difficulties was 34.3% (95% CI: 25.2%–47.9%%, *P* < 0.001) for the 8–9 h/d group and 41.2% (95% CI: 32.0%–54.5%, *P* < 0.001) for the <8 h/d group in the imputed data, consistent with the complete-case analysis (Supplementary Table S6). The confidence intervals overlapped substantially, and all estimates remained statistically significant, indicating that missing data did not substantially bias the results.

## Discussion

This study utilized a large-scale cluster random sampling approach to investigate the associations between preschool children's sleep duration and parental depressive symptoms in a socioeconomically disadvantaged region of western China, while concurrently examining the extent to which mental health outcomes may account for this association. Alarmingly, only 28.74% of preschool children in our study met recommended sleep duration (10–13 h/d), consistent with prior evidence from Beijing (29.5%) (44). This suggests that shorter sleep duration among preschool children is a pervasive issue across Chinese regions, rather than a phenomenon confined to specific areas. Indeed, a population-based study of Chinese preschoolers found that regional differences in sleep patterns were primarily in temporal placement rather than sleep duration (45). The rate of meeting recommended sleep duration in our sample is substantially lower than rates reported in high-income countries (e.g., 83.1%–94.0% in Australia, Belgium, Canada) (46–48). This stark disparity highlights a critical public health gap, underscoring the urgent need for targeted interventions to address insufficient sleep in early childhood.

Our findings revealed a robust and graded association between children's shorter sleep duration and parental depressive symptoms, with the strongest association observed in children sleeping <8 h/d (OR = 2.61, *P* < 0.001). This pattern aligns with previous evidence, including a multi-method actigraphy study showing that higher levels of paternal depressive symptoms are associated with fewer sleep minutes in school-aged children (16), and a longitudinal study demonstrating that maternal depressive symptoms independently predicted shorter-than-recommended sleep duration in preschoolers (15). Our findings extend prior evidence to a large understudied population in western China.

Linear regression showed that shorter sleep duration was associated with higher total difficulties scores in a graded manner, with the strongest increase observed in children sleeping <8 h/d. Logistic regression revealed that each one-point increase in total difficulties was associated with a 15% increase in the odds of parental depressive symptoms. Indirect effect analysis further revealed that children's mental health accounts for a substantial portion of the association between children's sleep and parental depressive symptoms. The observed pattern is consistent with a neurobiologically-informed model wherein sleep loss disrupts the top-down regulatory control of the prefrontal cortex (PFC) over the amygdala, potentially contributing to children's emotional and behavioral difficulties (49), which in turn may increase parental subsequent depression (26). However, the cross-sectional nature of our data precludes confirmation of such temporal pathways. A more complex, reciprocal dynamic may be at play over time: parental depression may heighten parenting stress, exacerbating children's emotional and behavioral problems (50), while child psychopathology is itself linked to disrupted sleep patterns (51, 52). Thus, children's sleep problems, mental health, and parental depression may be reciprocally interrelated. Our cross-sectional findings are consistent with this model but cannot confirm its directionality. Nonetheless, the present study extends prior literature by documenting this association in a large understudied population from western China.

Our stratified analyses identified housing situation, Hukou status, and parental smoking history as significant effect modifiers of the association between children's sleep duration and parental depressive symptoms, particularly in sleeping <8 h/d group. A clear ordered pattern emerged across housing situations, wherein the association between children's insufficient sleep and parental depressive symptoms was strongest among parents with mortgages, followed by renters, those cohabiting with relatives, and outright homeowners. This pattern aligns with levels of chronic financial strain—mortgaged homeowners bear significant debt burdens, while renters face housing instability and cost pressures, factors robustly linked to depression (53, 54), whereas cohabitation may introduce additional stressors such as overcrowding and complex family dynamics (55). Consistent with this, prior work has shown that lower socioeconomic status predicts shorter child sleep duration, an association mediated by home environment quality (56). Even outright homeowners, typically representing socioeconomic stability in China, faced significantly elevated risk, underscoring the inherent stress of managing children's sleep problems.

Concerning Hukou status, a unique household registration system shaping resource access in China (38), urban registrants exhibited a stronger association in sleeping <8 h/d group. While rural hukou households face economic and resource challenges (57, 58), urban settings concentrate environmental stressors like high living costs, competitive academic pressure (59), and weaker community support (60), amplifying the impact of children's insufficient sleep. For parental smoking history, ever-smokers showed markedly elevated risk of depression symptoms, particularly in <8 h/d group. This aligns with evidence linking smoking to pathogenesis (61) and elevated risk (62) of depression, as well as mechanistic studies showing that smoking impairs stress adaptation via hypothalamic-pituitary-adrenal (HPA) axis dysregulation (63). Additionally, parents with depressive traits may use nicotine for symptom relief, potentially perpetuating a cycle of mood deterioration (64, 65). Collectively, these findings suggest that socioeconomic disadvantage—reflected in housing-related financial strain, urban Hukou registration, and smoking history—may amplify the impact of children's shorter sleep on parental mental health, identifying these groups as high-risk subgroups for targeted interventions.

### Strengths and limitations

This study benefits from a large, regionally representative sample from an understudied region of western China, enhancing the generalizability of our findings to socioeconomically disadvantaged populations. Comprehensive adjustment for unique contextual confounders, such as Hukou status, housing stress, and multi-child family structures, strengthens the validity of our results. Furthermore, we identified high-risk subgroups for precision public health targeting. Finally, by examining the indirect role of children's mental health, this study provides important insights into the association between children's sleep duration and parental depressive symptoms.

Several limitations should be considered. First, the cross-sectional design precludes temporal sequence among children's insufficient sleep, mental health outcomes, and parental depressive symptoms. Second, sleep duration was parent-reported and may be subject to recall bias, especially among parents with depressive symptoms; future studies would benefit from objective measures like actigraphy. Third, although we adjusted for extensive covariates, residual confounding (e.g., parental sleep problems, parenting stress, family conflict) cannot be ruled out. We also did not assess perinatal depression, which may represent an additional unmeasured confounder. Finally, participants were recruited exclusively from preschoolers in western China, which may limit generalizability to other age or geographical or cultural contexts.

## Conclusion

The present study provides evidence for the association between preschool children's sleep duration and elevated parental depressive symptoms in western China, while also indicating that children's behavioral and emotional difficulties link this association. Moreover, parents facing housing insecurity, urban residency, or a history of smoking were identified as particularly vulnerable subgroups, highlighting the role of social determinants in this relationship. These findings highlight children's insufficient sleep as a significant family-level factor with public health implications.

## Data Availability

The raw data supporting the conclusions of this article will be made available by the authors, without undue reservation.
