# Supplementary material for: Preschool children's sleep duration and parental depressive symptoms in western China: role of children's mental health and socioeconomic factors
Source: Front Pediatr. 2026 Aug 11;14:1862244. doi: 10.3389/fped.2026.1862244 (PMC13503615; doi:10.3389/fped.2026.1862244)
Supplement: Supplementary file 1 [file Supplementaryfile1.docx]

**Table S1. Questionnaire items for covariates**

| **Variable** | **Questionnaire item** | **Response options** | **Analytic coding** |
| --- | --- | --- | --- |
| **Child gender** | Child's sex | Boy  Girl | – |
| **Child age** | Child's age in years | Continuous | – |
| **Primary parent gender** | Your sex | Men  Women | – |
| **Primary parent age** | Your age in years | Continuous | – |
| **Single-child status** | How many children are in your household? | One child  Two children  Three children  Four or more children | Yes（Single-child ）  No（Non-single-child） |
| **Marital status** | What is your current marital status? | Married  Unmarried  Divorced  Widowed  Separated  Cohabitating  Other | Married  Non-married |
| **Family annual income** | What was your total family income in the past year? (CNY) | <10,000  10,000–29,999  30,000–59,999  60,000–99,999  100,000–149,999  150,000–199,999  200,000–299,999  ≥300,000 | <30  30–99  ≥100  in thousands, CNY |
| **Parental education level** | What is your highest education level? | below primary school  primary school  Junior high school  High school (including vocational and technical secondary school)  Junior college  Bachelor’s degree  Master’s degree  Doctoral degree | Junior high school or less  High school or junior college  University degree or above |
| **Parental employment status** | What is your current employment status? | Employed  Unemployed | – |
| **Housing situation** | What is your current housing situation? | Owned outright  Owned with mortgage  Renting  Cohabiting with relatives | – |
| **Hukou status** | What is your household registration type? | Urban Hukou  Rural Hukou | – |
| **Smoking history** | Have you ever smoked? (former or current) | Yes  No | Ever-smoker  Never-smoker |
| **Alcohol use history** | Have you ever consumed alcohol? (former or current) | Yes  No | Ever-drinker  Never-drinker |
| **Sleep duration** | On average, how long did your child sleep per day (including daytime and nighttime sleep) during the past month? | <8 hours  8–9 hours  10–13 hours  >14 hours | <8h/d  8–9h/d  10–13h/d |

Note: – indicates variables used in the analysis as originally categorized.

**Supplementary Figure S1 Flowchart for sample selection**

**Supplementary** **Table S2 Variance inflation factor (VIF) analysis for multicollinearity assessment**

| **Variable** | **VIF** |
| --- | --- |
| Sleep duration | 1 |
| Total difficulties | 1.1 |
| Child gender | 1 |
| Child age | 1 |
| Primary parent gender | 2.4 |
| Primary parent age | 1.2 |
| Single-child status | 1.1 |
| Marital status | 1 |
| Family annual income | 1.2 |
| Parental education level | 1.5 |
| Parental employment status | 1.3 |
| Housing situation | 1.1 |
| Hukou status | 1.3 |
| Smoking history | 2.3 |
| Alcohol use history | 1.8 |

VIF values were calculated based on the fully adjusted model (Model III), including sleep duration, total difficulties, and all covariates listed in the Covariates section. Stepwise screening was applied with a threshold of 5. All variables exhibited VIF < 5, indicating no substantial multicollinearity.

**Supplementary Table S3 Variance components and intraclass correlation coefficients (ICC) from null models with kindergarten as random intercept**

| **Outcome** | **Between‑kindergarten variance** | **Within‑kindergarten variance** | **ICC** | **Model status** |
| --- | --- | --- | --- | --- |
| CES-D score | 0.073 | 44.65 | 0.0016 | OK |

ICC = between‑kindergarten variance / (between‑kindergarten variance + within‑kindergarten variance). For CES-D score, the between‑kindergarten variance was essentially zero, indicating no meaningful clustering by kindergarten. Therefore, standard regression models (without random effects) are appropriate for the primary analyses.

**Supplementary Table S4 Characteristics of study participants by sleep duration** **(N=21,366)**

|  | **Mean ± SD/ N (%)** | | |  | ***P-*value** |
| --- | --- | --- | --- | --- | --- |
|  | **≥10 h/d sleep duration**  **(N = 6,140, 28.74%)** |  | **<10 h/d sleep duration**  **(N = 15,226, 71.26%)** |  |  |
| **Child gender** |  |  |  |  | 0.163 |
| Boys | 3,225 (52.52%) |  | 7,837 (51.47%) |  |  |
| Girls | 2,915 (47.48%) |  | 7,389 (48.53%) |  |  |
| **Child age (years)** | 4.65 ± 0.89 |  | 4.88 ± 0.88 |  | <0.001 |
| **Primary parent gender** |  |  |  |  | 0.573 |
| Men | 1,452 (23.65%) |  | 3,656 (24.01%) |  |  |
| Women | 4,688 (76.35%) |  | 11,570 (75.99%) |  |  |
| **Primary parent age (years)** | 34.80 ± 4.60 |  | 34.73 ± 4.55 |  | 0.296 |
| **Single-child status** |  |  |  |  | <0.001 |
| Yes | 2,058 (33.52%) |  | 4,090 (26.86%) |  |  |
| No | 4,082 (66.48%) |  | 11,136 (73.14%) |  |  |
| **Marital status** |  |  |  |  | <0.001 |
| Married | 6,002 (97.75%) |  | 14,718 (96.66%) |  |  |
| Non-married | 138 (2.25%) |  | 508 (3.34%) |  |  |
| **Family annual income, in thousands, CNY** |  |  |  |  | <0.001 |
| <30 | 2,087 (33.99%) |  | 6,532 (42.90%) |  |  |
| 30–99 | 2,538 (41.34%) |  | 6,081 (39.94%) |  |  |
| ≥100 | 1,515 (24.67%) |  | 2,613 (17.16%) |  |  |
| **Parental education level** |  |  |  |  | <0.001 |
| Junior high school education or less | 1,307 (21.29%) |  | 4,434 (29.12%) |  |  |
| High school or junior college education | 2,745 (44.71%) |  | 6,731 (44.21%) |  |  |
| University bachelor education or above | 2,088 (34.01%) |  | 4,061 (26.67%) |  |  |
| **Parental employment status** |  |  |  |  | <0.001 |
| Employed | 4,598 (74.89%) |  | 11,044 (72.53%) |  |  |
| Unemployed | 1,542 (25.11%) |  | 4,182 (27.47%) |  |  |
| **Housing situation** |  |  |  |  | <0.001 |
| Owned outright | 3,483 (56.73%) |  | 8,094 (53.16%) |  |  |
| Owned with mortgage | 1,004 (16.35%) |  | 2,387 (15.68%) |  |  |
| Renting | 1,101 (17.93%) |  | 3,311 (21.75%) |  |  |
| Cohabiting with relatives | 552 (8.99%) |  | 1434 (9.42%) |  |  |
| **Hukou status** |  |  |  |  | <0.001 |
| Urban | 2,268 (36.94%) |  | 4,848 (31.84%) |  |  |
| Rural | 3,872 (63.06%) |  | 10,378 (68.16%) |  |  |
| **Smoking history** |  |  |  |  | 0.297 |
| Ever-smoker | 889 (14.48%) |  | 2,290 (15.04%) |  |  |
| Never-smoker | 5,251 (85.52%) |  | 12,936 (84.96%) |  |  |
| **Alcohol use history** |  |  |  |  | 0.014 |
| Ever-drinker | 948 (15.44%) |  | 2,560 (16.81%) |  |  |
| Never-drinker | 5,192 (84.56%) |  | 12,666 (83.19%) |  |  |
| **Parental depressive symptoms** |  |  |  |  |  |
| CESD | 10.01 ± 6.07 |  | 11.68 ± 6.77 |  | <0.001 |
| CESD <16 | 5,420 (88.27%) |  | 12,574 (82.58%) |  | <0.001 |
| CESD **≥**16 | 720 (11.73%) |  | 2,652 (17.42%) |  |  |
| **Total difficulties score** |  |  |  |  |  |
| Continuous | 9.40 ± 4.76 |  | 10.42 ± 5.08 |  | <0.001 |
| TDS ≤14 | 5,275 (85.91%) |  | 12,113 (79.55%) |  | <0.001 |
| TDS >14 | 865 (14.09%) |  | 3,113 (20.45%) |  |  |

Mean ± SD for continuous variables: the *P* value was calculated by Mann-Whitney U test; (%) for categorical variables: the *P* value was calculated by Fisher's exact tests. Abbreviations: SD, standard deviation; CNY, Chinese Yuan; h/d, hours/day; TDS, total difficulties score. Note: For descriptive purposes, sleep duration was dichotomized into ≥10 h/d and <10 h/d sleep duration.

**Supplementary Table S5 Sensitivity analysis using multiple imputation for missing age data: multivariate logistic regression results for the association between children’s sleep duration and parental depressive symptoms (N=22,609)**

| **Sleep duration (h/d)** | **Model I** | |  | **Model II** | |  | **Model III** | |
| --- | --- | --- | --- | --- | --- | --- | --- | --- |
|  | **OR (95% CI)** | ***P*-Value** |  | **OR (95% CI)** | ***P-*Value** |  | **OR (95% CI)** | ***P-*Value** |
| 10**–**13 | 1.0 [Reference] |  |  | 1.0 [Reference] |  |  | 1.0 [Reference] |  |
| 8**–**9 | 1.52 (1.40, 1.66) | <0.001 |  | 1.49 (1.37, 1.62) | <0.001 |  | 1.41 (1.29, 1.54) | <0.001 |
| < 8 | 3.16 (2.64, 3.78) | <0.001 |  | 3.08 (2.58, 3.69) | <0.001 |  | 2.56 (2.13, 3.07) | <0.001 |

Model I adjusted for none; Model II adjusted for child/parental age and gender; Model III adjusted for child/parental age and gender, single-child status, marital status, family annual income, education level, employment status, housing situation, Hukou status, smoking and alcohol use history.

Abbreviations: OR, odds ratio; CI, confidence interval; h/d, hours/day.

**Supplementary Table S6 Sensitivity analysis using multiple imputation for missing age data: indirect effect analysis of children’s sleep duration on parental depressive symptoms through children’s total difficulties (N=22,609)**

|  | **8–9 h/d sleep duration** | |  | **<8 h/d sleep duration** | |
| --- | --- | --- | --- | --- | --- |
|  | **Estimate (95% CI)** | ***P*-Value** |  | **Estimate (95% CI)** | ***P-*Value** |
| Total effect | 0.0382 (0.0285–0.0488) | <0.001 |  | 0.1392 (0.1017–0.1758) | <0.001 |
| Indirect effect (average) | 0.0131 (0.0108–0.0156) | <0.001 |  | 0.0573 (0.0470–0.0680 | <0.001 |
| Direct effect (average) | 0.0251 (0.0154–0.0359) | <0.001 |  | 0.0819 (0.0480–0.1150) | <0.001 |
| Proportion accounted for (average) | 34.3% (25.2%–47.9%) | <0.001 |  | 41.2% (32.0%–54.5%) | <0.001 |

Sleep duration of 10**–**13 h/d was set as reference. Adjusted for child/parental age and gender, single-child status, marital status, family annual income, education level, employment status, housing situation, Hukou status, smoking and alcohol use history.

Abbreviation: CI confidence interval
